# Taliglucerase alfa in the longterm treatment of children and adolescents with type 1 Gaucher disease: the Albanian experience

**DOI:** 10.3389/fped.2024.1352179

**Published:** 2024-02-23

**Authors:** Paskal Cullufi, Sonila Tomori, Virtut Velmishi, Agim Gjikopulli, Ilir Akshija, Aferdita Tako, Ermira Dervishi, Gladiola Hoxha, Marjeta Tanka, Erjon Troja, Mirela Tabaku

**Affiliations:** ^1^ Pediatric Department, University Hospital Center Mother Teresa, Tirana, Albania; ^2^ Statistics Department, University Hospital Center Mother Teresa, Tirana, Albania; ^3^ Radiology Department, University Hospital Center Mother Teresa, Tirana, Albania; ^4^ Pharmacy Department, University Hospital Center Mother Teresa, Tirana, Albania

**Keywords:** Gaucher disease, children, adolescent, Taliglucerase alfa, enzyme replacement therapy, efficacy, safety

## Abstract

**Introduction:**

Enzyme replacement therapy is already recognized as the gold standard of care for patients with Gaucher disease. Taliglucerase alfa is one of the three alternatives recommended for treatment of Gaucher disease in children and adults.

**Aim:**

This study aims to evaluate the long-term efficacy and safety of Taliglucerase alfa in children and adolescents with Type 1 Gaucher disease.

**Patients and methods:**

Over a six-year period, we monitored the efficacy of continuous treatment in 10 patients by assessing various parameters, including hemoglobin concentration, platelet count, liver and spleen volume, bone mineral density, glucosylsphingosine level, chitotriosidase activity, and growth parameters*.* Safety was evaluated by immunogenicity and adverse event monitoring.

**Results:**

The mean age of patients was 13.4 ± 3.6 years and the treatment duration was 60.24 ± 13.4 months. From baseline to end line the parameters change as follows: hemoglobin concentration improved from 12.7 (±1.3) to 14.6 (±1.5) and platelet count from 180 (±74) to 198 (±79). The spleen volume, was reduced by 46% (*p* = 0,007). The chitotriosidase activity decreased from 4,019.7 (±3,542.0) nmoles/ml/hr to 2,039.5 (±1,372.2) nmoles/ml/hr (46% reduction). Glucoylsphingosine level dropped from 119.2 (±70.4) ng/ml to 86.2 (±38.1) ng/ml, indicating a reduction of 28%. Bone mineral density Z-score, improved from −1.47 (±1.76) to −0.46 (±0.99) (69.7% reduction). Out of the 1,301 total administrations, our patients reported only 37 (2.8%) infusion-related adverse events which were mild and transitory.

**Conclusion:**

Taliglucerase alfa exhibits good efficacy and a safe profile in the treatment of children and adolescents with Type 1 Gaucher disease.

## 1 Introduction

Gaucher disease (GD), OMIM #230800, ORPHA355) is an autosomal recessive genetic disorder affecting approximately 1 in 40,000 to 1 in 100,000 of the global population (1–3). It is a lysosomal storage disorder caused by pathogenic biallelic variants in the *GBA1* gene, which encodes the lysosomal enzyme acid beta-glucocerebrosidase. The *GBA1* pathogenic variants lead to a deficiency in the glucocerebrosidase enzyme, resulting in the accumulation of glucosylceramide in the lysosomes of macrophages in various organs, notably in the reticuloendothelial system, responsible for most clinical manifestations (4–7). The two main phenotypes of this illness are the non-neuronopathic form (GD type 1), the most frequent, accounting for about 85%–90% of cases, and the neuronopathic phenotype (GD types 2 and 3). However, a continuum of phenotypes can be observed, ranging from asymptomatic or mild to lethal perinatal forms (4, 8, 9). The significant phenotypic heterogeneity is partly explained by the numerous (>860) pathogenic variants found in the *GBA1* gene to date, as well as by several genetic, epigenetic, and environmental factors (3, 9).

Enzyme replacement therapy (ERT) is already recognized as the gold standard of care for GD patients (10–12). Three ERT drugs are available for the treatment of GD patients: Imiglucerase (Sanofi/Genzyme); Velaglucerase alfa (Takeda-Shire); and Taliglucerase alfa (Pfizer-Protalix). Taliglucerase alfa (TGa) is produced in carrot cells and is the first recombinant therapeutic protein produced in a plant cell expression system to be approved for use in humans by the American Food and Drug Administration (10, 11, 13). TGa differs from native glucocerebrosidase by 2 amino acid residues at the C-terminus and up to 7 amino acid residues at the N-terminus (5). Imiglucerase differs from native glucocerebrosidase at amino acid residue 495, where it has a histidine instead of an arginine in the C-terminus. Velaglucerase alfa has an identical secondary structure to native glucocerebrosidase (5). Taliglucerase alfa does not require additional steps to create glycan structures necessary for cellular uptake by Gaucher cells (14–16). As it is plant-derived, it can cause more adverse reactions than mammalian-derived enzymes (16). TGa appeared to have similar safety and efficacy profiles compared to imiglucerase and velaglucerase alfa (5).

Taliglucerase alfa is recommended for treating adults and children with GD1, and in some countries, including ours, it is also used to treat the hematologic and visceral symptoms of type 3 Gaucher disease (10–13). Our research study aims to evaluate the long-term results on safety and safety of TGa in children and adolescents with GD1.

## 2 Material and methods

This prospective study was conducted at the Gaucher Unit of the “Mother Teresa” University Hospital in Tirana, Albania. The period of data collection was January 2016–December 2021. The study obtained approval from the national ethical committee. All patients or their parents, in the case of minors, before inclusion in the study, provided signed informed consent forms.

### 2.1 Patients

A total of 10 children and adolescents treated with Type 1 GD, treated with TGa for at least 2 years, were enrolled and monitored for a period of 6 years. Upper age limit for adolescents is defined according to the American Academy of Pediatrics, which categorizes adolescence as 11–21 years of age, further divided into early adolescence (ages 11–14), middle adolescence (ages 15–17), and late adolescence (ages 18–21) (17). At the baseline, we collected epidemiologic data for all patients, including age, gender, height, weight, race, ethnicity, and genotype.

### 2.2 Efficacy assessment

To evaluate efficacy, the following parameters were monitored: Hemoglobin concentration (HGB) and platelets count (PLT) were evaluated once a year. Spleen and liver volumes were evaluated once a year by Magnetic Resonance Imaging (MRI) and Computed Tomography (CT scan) in some cases (due to patient refusal of MRI or its unavailability). Volumes were expressed in multiples of the normal volume (MN). The normal liver volume was calculated as 25 ml/kg/weight, whereas the normal spleen volume was 2 ml/kg/weight. Bone mineral density (BMD) was evaluated by Dual x-ray Absorptiometry (DXA) performed for the vertebral spine once a year. Chitotriosidase activity was evaluated every six months using DBS cards sent to the Child Institute of Health in Athens, Greece. The reference value is <150 nmoles/ml/hr. We have not done genetic testing for eventual mutations of the CHIT gene, since our patients had high levels of chitotriosidase activity at the time of diagnosis. Glucosylsphingosine (Lyso-GB1) was assessed every three months using Dried Blood Spot (DBS) cards sent to Centogene Laboratory in Rostock, Germany. The normal reference value for Lyso-GB1 is <6.8 ng/mol. Growth was assessed by measuring and recording weight and height every 12 months, while puberty development was evaluated annually using the Tanner Scale. Assessments stopped once the patient reached stage 5 of the Tanner scale.

### 2.3 Safety assessment

Taliglucerase alfa safety was evaluated by immunogenicity, which was determined by looking for anti-taliglucerase alfa antibodies (ADA), and monitoring of adverse event (AE). Blood samples for ADA were collected every six months and subsequently sent to Covance Laboratory in Indianapolis, USA.

### 2.4 Statistical analyses

Categorical variables were represented as frequency and percentages, and descriptive continuous variables were represented as means and standard deviations. The student's *T*-test was performed to test changes from baseline. Statistical analysis was conducted using IBM SPSS Statistics 26.0 software.

## 3 Results

### 3.1 Epidemiologic data

As depicted in Table 1, there are 10 children and adolescents enrolled in this study, comprising 7 boys and 3 girls, all of Caucasian race and Albanian ethnicity. The mean age of patients at the initiation of treatment with TGa was 13.4 ± 3.6 years, ranging from 6.9 to 16.5 years old. Eight of them had previously undergone treatment with Imiglucerase, while two were treatment-naïve patients.

**Table 1:** Epidemiologic data.

Age	
Mean ± SD	13.4 ± 3.6 years
Range	6.9–16.5 years
Gender	
Male	3 (30%)
Female	7 (70%)
Race	White (100%)
Ethnicity	Albanian (100%)
Genotype	
*p.Asn409Ser/p.Asp448His:p.His294Gln*	7 (70%)
*p.Asn409Ser /p.Ser146Leu*	1 (10%)
*p.Asn409Ser /p.Ser146Leu*	1 (10%)
p.Asn409Ser/p.Arg86*	1 (10%)
Patients treatment status	
Switched	8 (80%)
Naïve	2 (20%)
Treatment duration (months)	
Mean ± SD	60.24 ± 13.4
Range	41.2–72
Infusions per month/patient	
Mean	1.84
Dosage (mean)	
Baseline	55.23 UI/kg weight
Endline	47.20 UI/kg weight

The starting dose for switched patients was identical to the last dose of imiglucerase, whereas for treatment-naïve patients, according to our National Gaucher Disease protocol, the starting dose was 60 UI/kg weight. The mean dose of TGa at baseline was 55 UI/kg/weight, and by the end, it had decreased to 47 UI/kg/weight, representing a 15% reduction. The average duration of treatment was 60.24 ± 13.4 months, ranging from 41.2 to 72 months.

Only one patient stopped receiving TGa treatment, due to personal reasons unrelated to health or medical concerns. The total number of drug infusions administered was 1,301, averaging 1.84 drug infusions per month per patient, as opposed to the normal rate of 2.1 per month (26 infusions/year). This discrepancy was attributed to temporary constraints in the drug supply as well as difficulties accessing the Gaucher Unit due to COVID-19 pandemic-related limitations.

Among the patients, seven of them exhibited the genotype *p.Asn409Ser/p.Asp448His:p.His294Gln*, while three others had the genotype *p.Asn409Ser/other* (Table 1).

### 3.2 Blood parameters

**Figure 1:**
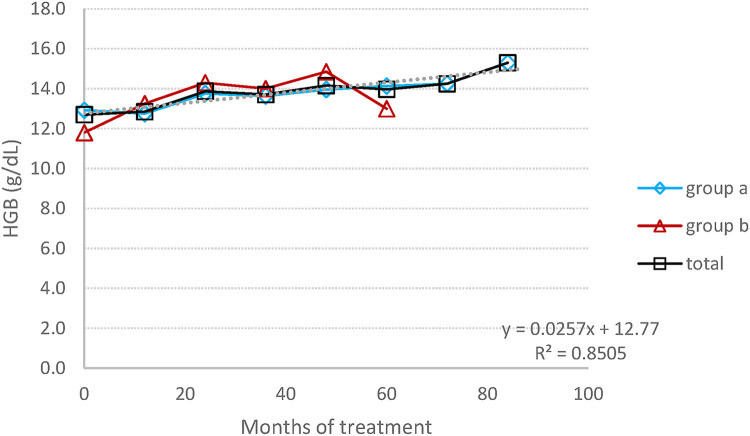
HGB concentration: the average change in the concentration of HGB during the entire follow-up period.

**Figure 2:**
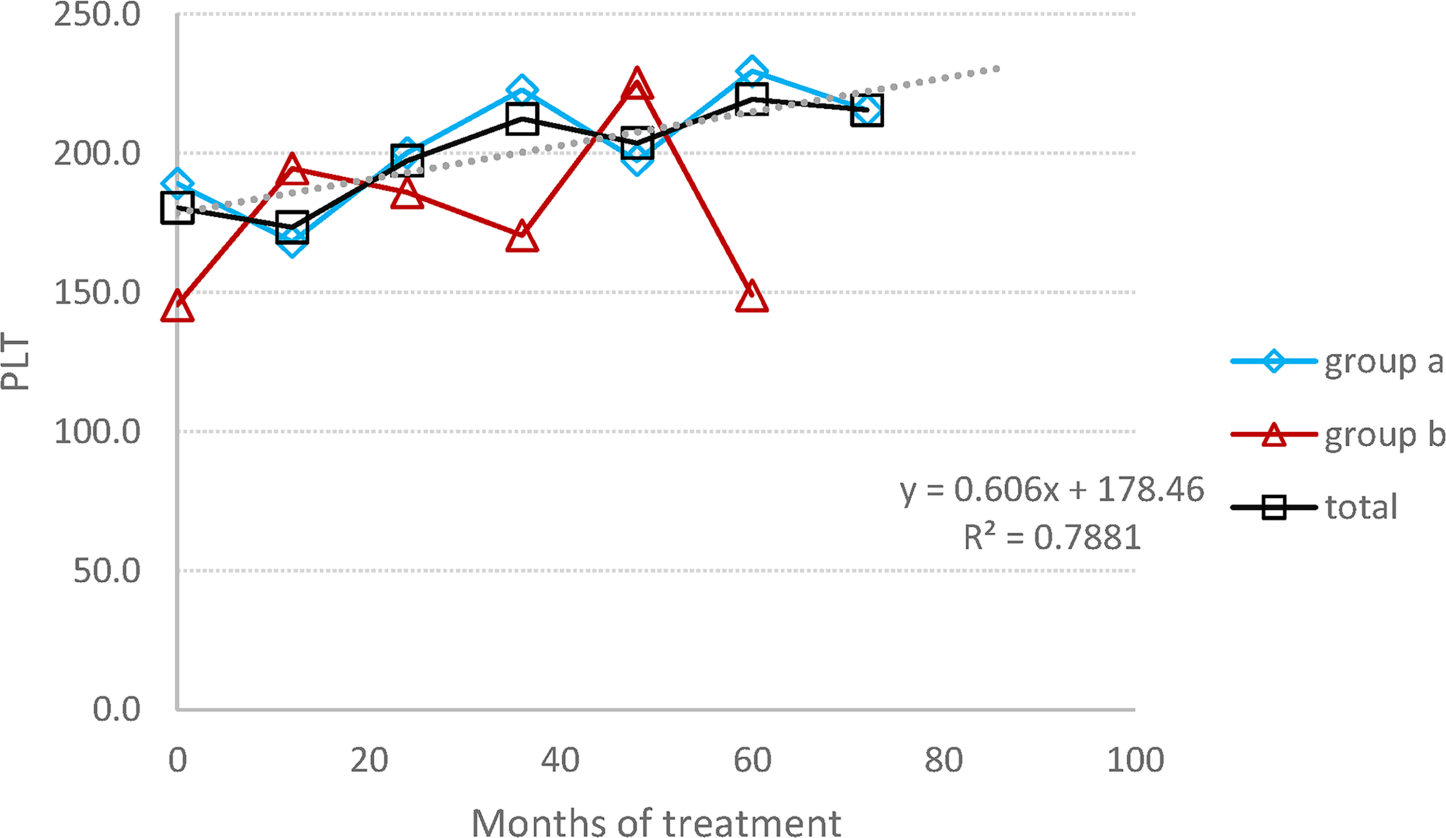
PLT count: the average change in the PLT number during the entire follow-up period. (**A**) Switched patients; (**B**) treatment-naïve patients and all 10 patients.

The mean average of HGB concentration remained within the normal range, with a significant increase graphic (Figure 1), changing from 12.7 (±1.3) at baseline to 14.6 (±1.5) at the endpoint (*p* = 0.02), corresponding to a 13% rise. The mean platelet count, despite individual variations in some patients (three patients had a PLT level between 120,000 and 150,000 at some time point), has practically remained stable, within normal range, increasing from 1,180 (±74) at baseline to 198 (±79) at the endpoint (*p* = 0.72), corresponding to a 6% change (Figure 2).

### 3.3 Spleen and liver volumes

**Figure 3:**
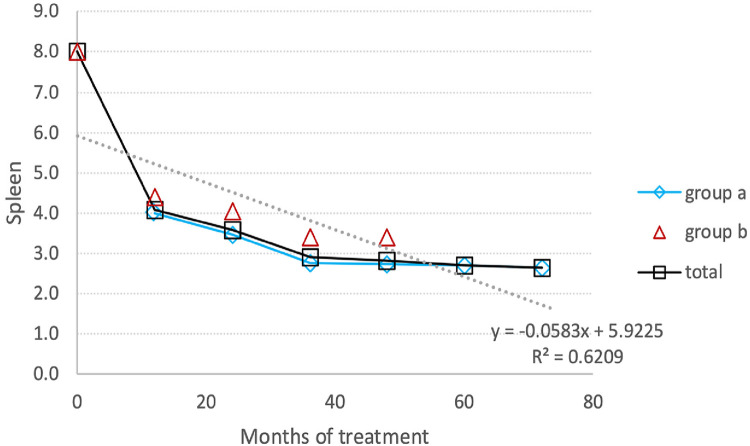
Spleen reduction: the average reduction in spleen volume during the entire follow-up period. (**A**) Switched patients; (**B**) treatment-naïve patients and total all patients.

Spleen volumes exhibited a noteworthy decrease of approximately 45%, dropping from −1.47 (±1.76) MN to −0.46 (±0.99) MN, indicating a 69.7% reduction (Figure 3). In treatment-naïve patients, the reduction of the spleen volume was more pronounced than in switched patients, decreasing from 8 MN to 3.4 MN, corresponding to a 58% reduction.

The liver volume was essentially normal at baseline, with two patients having liver volumes of 1.2 and 1.5 MN. By the end of the study, all patients’ liver volumes had returned to normal, and there were no signs or symptoms of liver cirrhosis.

### 3.4 Chitotriosidase activity

**Figure 4:**
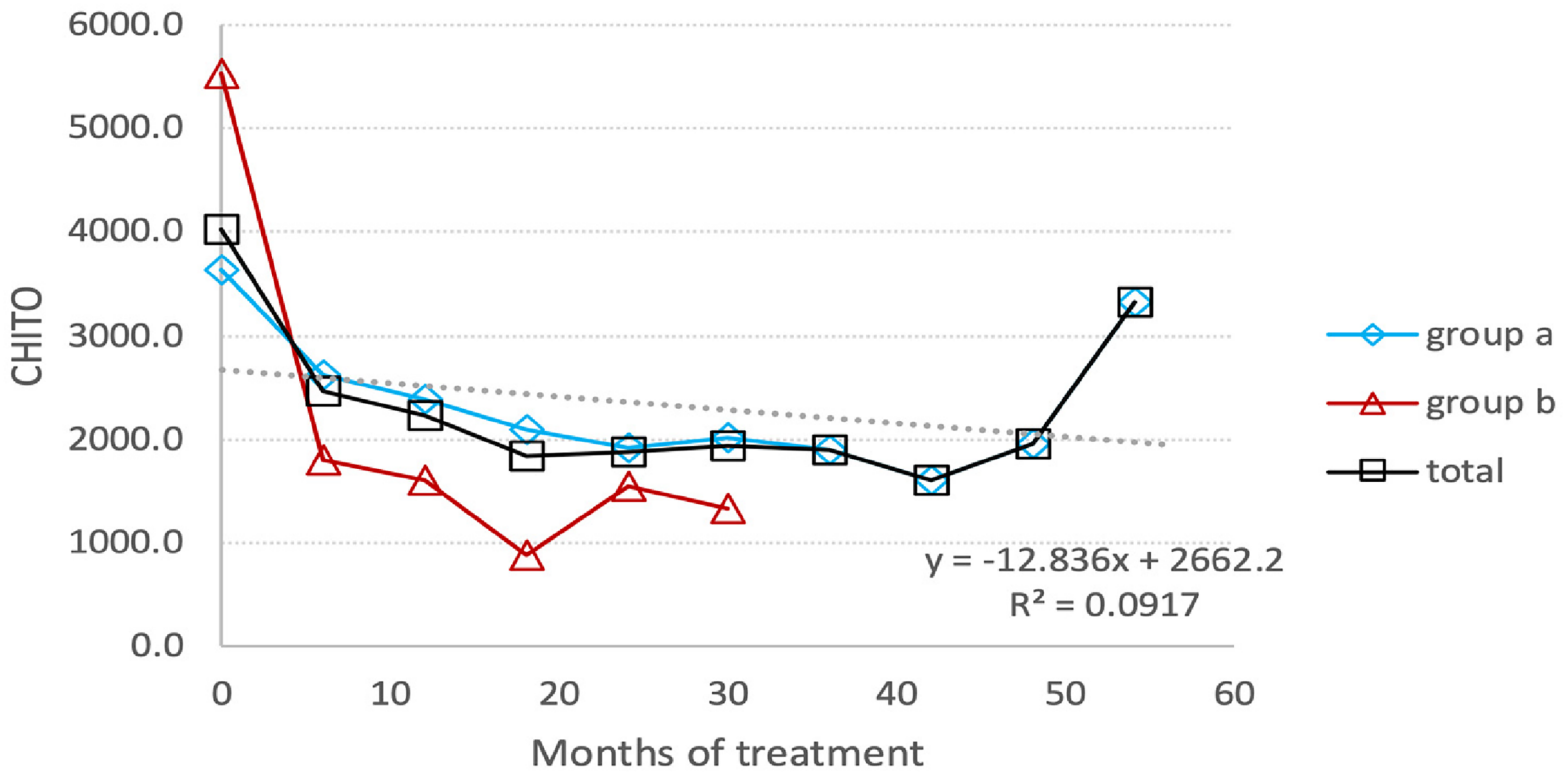
Chitotriosidase activity: the average reduction in the activity level of chitotriosidase under TGa treatment throughout the entire follow-up period. (**A**) Switched patients; (**B**) treatment-naïve patients and total all patients.

The chitotriosidase activity was measured every six months for the first 49.5 ± 2.77 months for switched patients and 19.5 ± 9.2 months for treatment-naïve patients. Afterward, chitotriosidase activity was not utilized for monitoring Gaucher patients. The mean change in chitotriosidase activity from baseline to endline was as follows: it decreased by 46% for all patients, from 4,019.7 (±3,542.0) nmoles/ml/hr to 2,039.5 (±1,372.2) nmoles/ml/hr. Switched patients experienced a decrease of 49%, going from 6,710.67 nmol/ml/hr to 4,089.78 nmol/ml/hr, while treatment-naïve patients had an 81% decline from 7,374 nmol/ml/hr to 1,420 nmol/ml/hr. The last measurement of Chitotriosidase activity shows an increase level (Figure 4). In fact, this represents the value for a single patient, which results to be higher than the average value of the entire group in the previous measurement. But, even in this patient we have a decrease in chitotriosidase activity from 10,725 nmol/ml/hr (baseline) to 3,330 nmol/ml/hr (last measurement).

Notably, in a 9-year-old girl, an increase in chitotriosidase activity was observed, rising from 792 nmol/ml/hr at baseline to 1,192 nmol/ml/hr and 2,427 nmol/ml/hr in two consecutive measurements, following an initial decrease in activity. At the same time that chitotrisosidase activity reached 2,426 nmoles/Ml/hr, the level of lyso-Gb1 dropped from 112 ng/ml to 82 ng/ml.

### 3.5 Glucosylsphingosine (lyso-Gb1)

**Figure 5:**
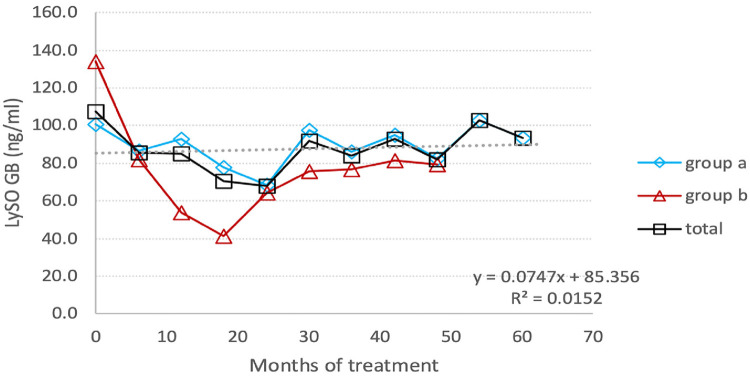
Individual Lyso-GB1 curves : the change in the level of Lyso-Gb1 depending on the treatment with TGa, during the entire follow-up period. The interrupted line indicates the mean values of Lyso-GB1.

The lyso-Gb1 level was monitored every three months, and Figure 5 presents the results for switched patients, treatment-naïve ones, and the entire group. At the endpoint, the group's total lyso-Gb1 levels decreased from 119.2 (±70.4) ng/ml at baseline to 86.2 (±38.1) ng/ml, indicating a 28% drop.

#### 3.5.1 Switched patients

**Figure 6:**
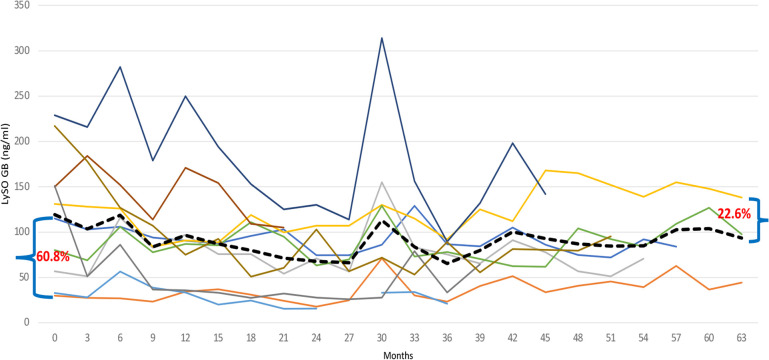
Individual Lyso-GB1 curves: the change in the level of Lyso-Gb1 depending on the treatment with TGa, during the entire follow-up period. The black line indicates the Lyso-GB1 means.

The most significant reduction, from a mean of 103.05 ng/ml to a mean of 60.95 ng/ml (40.8%), occurred after 24 months of monitoring lyso-Gb1. At this point, there was an involuntary treatment break lasting about three consecutive months, which was associated with a prominent increase in lyso-Gb1 levels, more than twice as high as the previous measurement. After the restart of TGa treatment, the reduction in lyso-Gb1 levels continued, albeit at a lower rate of 22.8%, resulting in a decrease from a mean of 131.27 ng/ml to 102.7 ng/ml (Figure 6). In this group, we found out that lyso-Gb1 levels were lower in five patients harboring the *p.Asn409Ser* mutation linked to the double allele mutation *p.Asp448His;His294Gln* than in three patients carrying the *p.Asn409Ser*/others genotype. The mean lyso-Gb1 levels were 69.88 ng/ml and 158.3 ng/ml at the baseline measurement and 67.74 ng/ml and 121.37 ng/ml at the final measurement, respectively, showing a roughly twofold decrease at both time points.

#### 3.5.2 Naïve patients

Naïve patients exhibited a decline in lyso-Gb1 levels during the follow-up period, with a reduction from 159.7 ng/ml at baseline to 80 ng/ml at the last measurement, corresponding to a 50% decrease. The reduction rate trend was as follows: 42.1% at 6 months after TGa treatment; about 70% at the end of the first year, and 77.5%. at the end of the second year. Afterwards, the Lyso-GB1 reduction trend was affected by treatment break period, resulting in increase levels of this biomarker (Figure 5).

### 3.6 Bone mineral densitometry (BMD)

**Figure 7:**
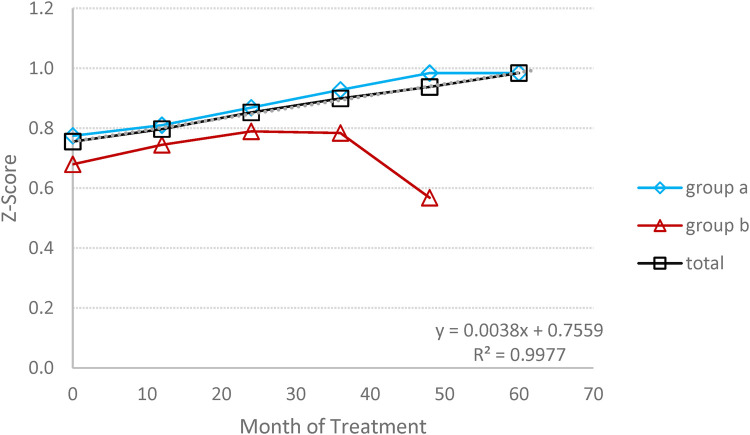
Bone mineral densitometry (BMD): the average changes in BMD expressed by Z score, during the follow-up period. (**A**) Switched patients; (**B**) treatment-naïve patients and total all patients.

At baseline, three patients exhibited Z-score <−2.5, and three others showed a Z-score between −1 to −2.5. All patients, with the exception of one, demonstrated a significant improvement in BMD (Figure 7). The group's bone mineral density as defined by the Z-score, increased by 69.7%; from −1.47 at baseline to −0.46 at the endpoint (*p* = 0.161).

Notably, the patient who did not show improvement in BMD exhibited consistently high and persistent Lyso-Gb1 levels >150 ng/mol, throughout the study, even though other parameters were normalized.

### 3.7 Growth

Out of the 10 patients, 7 had already reached puberty upon joining the study. The weight and height of these patients showed improvement, and they all fall within normal ranges according to WHO percentile charts. However, one patient exhibited a weight below the normal percentile, while the height remained within the normal range.

Only two girls began treatment at the ages of 6 and 7 years old. According to the Tanner Scale, both females experienced typical puberty development and achieved a normalized weight with TGa. One girl experienced her first menarche at 13 years old, and the other, at 11 years old, is at stage 3 of Tanner puberty. However, it's worth noting that the height of one of the girls was below 2 standard deviations.

## 4 Safety

### 4.1 Adverse events

Out of the 1,301 total administrations, our patients reported only 37 infusion-related adverse events, indicating a low incidence of 2,8%. These adverse events typically occurred during the initial infusions and were generally mild and temporary. The most frequently observed types of infusion-related AEs included urticaria (hives), cough, sneezing, pruritus, nausea, and lip edema reported by three of our patients (30%). The most common adverse events unrelated to drug infusion were rhinopharyngitis (36%), bone pain (15.7%), anxiety disorders (10.5.%), covid19 infection (10.5.%) (Table 2).

**Table 2:** Adverse events, infusions related and no infusion related.

AEs -infuions related	AEs number	%	AEs-no infusions related	AEs number	%
Urticaria	17	45.9	Rhinopharyngitis	7	36.8
Edema (lips)	5	13.5	Bone pain/arthralgia	3	15.7
Cough	4	10.8	Anxiety disorders	2	10.5
Sneeze	4	10.8	Covid 19	2	10.5
Pruritis	3	8.1	Others	5	26.3
Others	4	10.8			
Total AEs	37	100	Total	19	100

### 4.2 Immunogenicity

In terms of immunogenicity, blood samples collected during the first three years of treatment were tested. IgG anti-taliglucerase alfa antibodies (ADA) were identified in only one patient (10%), and at a low titer. At first, this patient showed a negative result in the initial year of treatment. However, after one year of TGa, he tested positive with a titer of 70. Subsequent measurements revealed titers of 74 and 59 in the following two years. Interestingly, this patient experienced three infusion-related adverse events: lipedema, urticaria, sneezing, and pruritus.

## 5 Discussion

Since its introduction in the 1990s, enzyme replacement therapy has become the standard of care for patients with Type 1 Gaucher Disease (GD) (3, 11, 12). Both children and adults, whether naïve or switched patients, have exhibited clinically and statistically significant improvements in the major clinical characteristics of GD1 under the treatment of TGa.

The results of our study indicate that all follow-up parameters, such as hemoglobin concentration, platelet count, liver and spleen volume, remained stable or improved, a consistent finding in other studies (10, 12).

Chitotriosidase activity was found to decline significantly over the study's course, showing the drug's efficacy. However, in a 9-year-old female patient, chitotriosidase activity changed from a decreasing profile in the first measurement from baselines to an increase of 50% over a two-time point course. This increase in chitotriosidase activity occurred without any episode of clinical deterioration or dose change. The coadministration of a corticosteroid is the most likely cause because the child was being treated for bronchial asthma, and corticosteroids have been shown by Van Duessen to affect chitotriosidase levels (18). Actually, the increase of chitotriocidase activity was accompanied by a decrease in the level of lyso-GB1, which further strengthens the idea of corticosteroids’influence in chitotriocidase activity.

Lyso-Gb1, the deacylated form of glucosylceramide, appears to be the most accurate biomarker for monitoring patients with Gaucher Disease. Its level correlates with the severity of the disease and significantly decreases under enzyme replacement therapy (7, 19–21). The reduction is higher in treatment-naïve patients than in switched ones. A reduction of 70% is observed in our two treatment-naïve patients during the first year of treatment, seemingly higher compared to other reports (19–21). Rather than the drug itself, the differences are probably related to the different characteristics of our patients, such as age, the severity of the disease, genotypes, and the limited number of patients. Switched patients in our study display a reduction of 41% after 22 months of taliglucerase alfa therapy. As previously mentioned, during that time, our patients had an involuntary break from treatment for about three months. Their lyso-Gb1 level significantly increased by more than twofold from the last measurement done before the break, regardless of the fact that neither their clinical symptoms nor their hematologic parameters had changed (22). This fact demonstrates that monitoring lyso-Gb1 enables the flagging of absent/insufficient treatment before clinical consequences arise (22).

The genotype *p.Asn409Ser/p.Asp448His; His294Gln* is the most frequent in the Albanian Gaucher population, accounting for more than 60% of GD patients, and it is generally associated with a mild to moderate genotype (23). Regarding the correlation between genotypes and lyso-Gb1 levels, we note that patients with the genotype *p.Asn409Ser/p.Asp448His;His294Gln* had lower levels of lyso-Gb1 at both baseline and the last measurement, compared to those with the genotype *p.Asn409Ser*/other.

Bone disease poses a significant challenge in GD1 treatment, with osteopenia and osteoporosis frequently observed in Gaucher patients across different ages and genders. Reduced bone mineral density (BMD) can be detected as early as 5 years of age, becoming more pronounced during adolescence (24–26). Various studies have demonstrated a positive effect of ERT therapy on the frequency of bone pain, bone crisis, and BMD (10, 26). Most clinical parameters, including BMD, have been reported to normalize or nearly normalize in children receiving ERT within 8 years (27). Our data align with this, as the mean Z-score for BMD at the endpoint results in a significant change of about threefold compared to the baseline.

However, despite this overall improvement, one of our patients reported occasional bone pain, experiencing a relatively severe episode of back pain lasting nearly a week. Our investigation found no evidence supporting other causes for this event.

Another patient exhibited persistence of BMD Z scores at approximately −2, along with a high lyso-Gb1 level of more than 150 ng/ml during the course of treatment. The persistent high level of lyso-Gb1 could potentially explain the inadequate treatment response in improving BMD, as Lyso-Gb1 may actively contribute to low mineral density by interfering with normal osteoblast function (7), as reported by Dekker. Additionally, the patient's genotype *p.Asn409Ser/p.Ser146Leu*, which is uncommon in our population, appears to be associated with a relatively severe phenotype.

Under the TGa treatment, all but one of our patients exhibited normalized growth and development parameters. A young girl measured at -2SD in height at the final assessment. It is plausible that the observed deviation in height for this girl could be attributed to concomitant pathology, specifically the solitary maxillary incisor syndrome. This condition might have influenced her growth pattern and contributed to the observed height measurement of less than -2SD. It is important to consider such additional factors when interpreting growth and development in patients, as comorbidities can play a significant role in shaping their overall health outcomes.

TGa has a good safety profile when treating children and adolescents (28, 29). Our study supports this through the very low incidence of infusion-related adverse events and the moderate severity and transient nature of these AEs. On the other hand, the development of anti-drug antibodies is commonly observed with recombinant therapeutic proteins. In GD patients, antitaliglucerase alfa antibodies have also been reported, but their presence has no impact on the effectiveness of ERT (28, 29). In our Gaucher unit, we also observed that anti-drug antibodies affected the efficacy of the drug in an adult, likely due to the patient's inherited immunological dysregulation, as the patient's family has a strong history of autoimmune/inflammatory disease (30).

Conclusion: Our long-term study demonstrates that TGa exhibits good efficacy and a safe profile in the treatment of children and adolescents with Gaucher disease.

## Data Availability

The raw data supporting the conclusions of this article will be made available by authors, without undue reservation.
